# Comparative evaluation of enamel surface roughness after debonding using four finishing and polishing systems for residual resin removal—an in vitro study

**DOI:** 10.1186/s40510-019-0269-x

**Published:** 2019-05-06

**Authors:** Priyanka Shah, Padmaja Sharma, Santosh Kumar Goje, Nikita Kanzariya, Maitry Parikh

**Affiliations:** Department of Orthodontics and Dentofacial Orthopedics, K.M. Shah Dental College & Hospital, Sumandeep Vidyapeeth, Piparia, Vadodara, Gujarat 391760 India

**Keywords:** Stainbuster, Enamel roughness, Scanning electron microscopy, Enhance and Pogo polisher, One gloss, Soflex discs

## Abstract

**Background:**

Orthodontic bonding and debonding procedures involve risk of damaging the enamel surface and changing its original morphology. The rough surface inhibits proper cleaning, invites plaque deposition, bacterial retention, and stain formation thus dampening the esthetic appearance of the teeth. Restoring the enamel to its original morphology is a challenge. Researches on better adhesive removal methods which can effectively remove the residual resin and restore it best to its original form are continuing till date. No study has compared four contemporary finishing systems for their efficiency on a single platform.

**Aim:**

The objective of this study is to evaluate and compare enamel surface roughness after debonding using four different finishing and polishing systems.

**Material and methods:**

Adhesive resin was removed from the buccal surface of 88 premolars after debonding with 4 groups. It included 22 teeth per group: group 1—One gloss system; group 2—Enhance finishing and polishing system; group 3—fiber reinforced stainbuster bur; and group 4—Soflex discs with wheels. Roughness was measured quantitatively and qualitatively with the help of surface roughness tester and scanning electron Microscope (SEM) respectively.

**Results:**

No significant difference was found in baseline roughness in four groups. Highest post-polishing roughness was observed in Soflex group (4.62 μm) followed by One gloss system (3.36 μm), Enhance system (3.17 μm), and stainbuster bur (1.99 μm) (*p* value < 0.01).

**Conclusion:**

Stainbuster bur created the smoothest enamel surface that was close to the natural enamel followed by Enhance system, One gloss system, and Soflex disc and wheels.

## Introduction

Debonding is a procedure of removing orthodontic attachments along with the entire residual adhesives from the surface of enamel following orthodontic treatment completion. The objective of the orthodontists should be to restore the surface of enamel as closely as possible to natural enamel without inducing iatrogenic injury and with minimal loss of enamel structure [1].

The surface structure of natural enamel has micro-roughness in the range of 0.59 to 0.66 μm [2]. Bonding of brackets on enamel involves surface etching, application of primer, and adhesive. All these steps involve the risk of damaging the enamel surface and changing its original morphology. Upon completion of orthodontic treatment, the debonding procedure followed by residual resin removal further damages the enamel, producing scratches, cracks, grooves, removal of fluoride-rich external enamel layer, and increasing the enamel roughness. The rough enamel surface inhibits proper cleaning and thus invites plaque deposition, bacterial retention, stain formation, and thus dampening the esthetic appearance of the teeth. Restoring the enamel to its original morphology is a challenge [3].

A variety of mechanical methods have been proposed to achieve satisfactory resin removal with least possible enamel damage following bracket debonding. These include band removing pliers, hand scalers, ultrasonic cleaning, intraoral sandblasting, sandpaper discs, diamond burs, stainless steel burs, rubber cups, tungsten carbide burs (fine or super fine grit, low or high speed, various flutes), lasers, and composite burs [4, 5].

Numerous methods are available to assess the enamel damage such as scanning electron microscopy (SEM), stereo microscopy, contact profilometry, a non-contact white light 3D profilometry, or atomic force microscopy (AFM) [4, 6].

In contemporary orthodontic practice, four finishing and polishing systems are being widely used. The One gloss complete system (Shofu Dental Corporation, Japan) uses a high concentration of aluminum oxide with silicone as a binder [7]. The Enhance finishing and Pogo polishing system (Dentsply, Milford, USA) is also widely used and is made up of polymerized urethane dimethacrylate resin, aluminum oxide, silicon dioxide, and fine diamond powder [8].

A new innovative composite bur enriched with zircon-rich glass fiber has gained attention. This fiber-reinforced composite bur, Stainbuster (Abrasive Technology Inc., Lewis Centre, Ohio), is also studied and compared with other systems though not extensively and with mixed results [9].

Lastly, the 3M Soflex system (3 M ESPE, St. Paul, MN, USA) which includes finishing discs and the most recently introduced Soflex spiral wheels are also being used by orthodontists. The discs are made up of aluminum oxide particles from coarse to superfine (50 to 80 μ), whereas the spiral wheels are made up of diamond particles impregnated in thermoplastic elastomer [10, 11].

There is no study wherein all these four contemporary finishing and polishing systems have been compared at one platform to assess the extent of enamel surface roughness after finishing and polishing. Thus, the aim of our study is to evaluate and compare enamel surface roughness after debonding using Shofu One gloss, Dentsply Enhance finisher and Pogo polisher, Fiber reinforced Stainbuster bur, and 3M Soflex discs and spiral wheels for residual resin removal.

## Methods

The study was carried out in the Department of Orthodontics, K. M. Shah Dental College & Hospital, Vadodara in collaboration with Ahmedabad Engineering Research Institute, Ahmedabad and Metallurgical & Material Engineering Department, Faculty of Technology and Engineering, The M.S. University of Baroda, Vadodara.

Selection criteria included extracted premolars with intact buccal surface. Premolars with (1) carious lesions, (2) restorations, (3) visible cracks, and (4) hypoplasia were excluded.

**Fig. 1:**
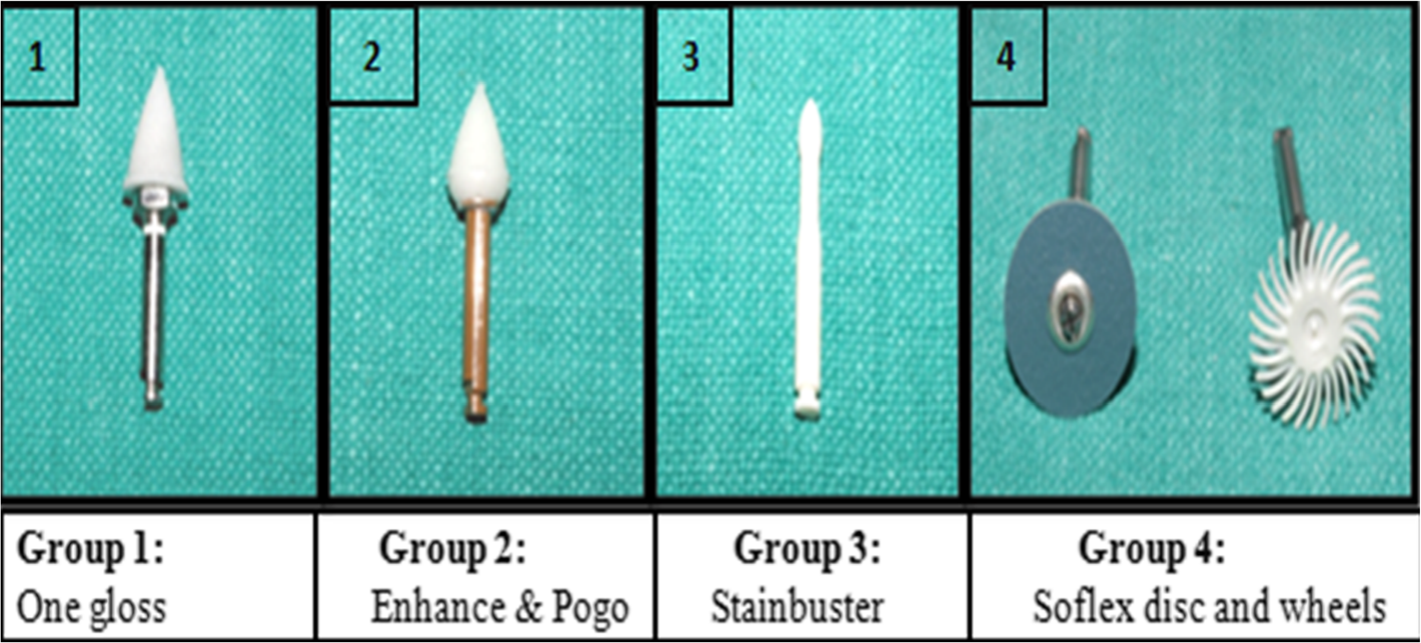
Four groups used in the study

Assuming that the significant difference required between two groups in relation to mean roughness is 0.33 μm based on the values obtained from previous study [4]. Sample size in this study was calculated using significance level of 0.05 and power of 80% to detect meaningful differences among mean values of four groups. It showed that minimum of 88 samples (22 per group) were required. Figure 1 illustrates four groups. Table 1 describes four groups used for roughness check.

**Table 1:** Description of four groups

Groups	Resin removal methods	Company	Number of teeth
1	Shofu one gloss complete set	Shofu Dental Corporation, Japan	22
2	Enhance Finishing Kit	Dentsply, Milford, USA	22
3	Fiber reinforced composite bur	Stainbuster, Abrasive Technology Inc., Lewis Centre, Ohio	22
4	Soflex finishing disc and spiral wheels	3 M ESPE, St. Paul, MN, USA	22

The sample of 88 premolars extracted for orthodontic purpose was procured as per the inclusion criteria. The teeth were washed under running water to cleanse the soft tissue remnants and stored in 0.1% thymol for disinfection. Then the teeth were mounted in Plaster of Paris leaving the crown of the teeth visible.

The 88 mounted teeth were randomly divided into 4 groups of 22 each for residual resin removal. The randomization was done using computer randomization method (Research Randomization Program).

Buccal surfaces of teeth on the area where bracket is bonded were evaluated with the help of surface roughness tester (Baseline roughness data). Two measurements for each specimen were measured and mean was calculated.

**Fig. 2:**
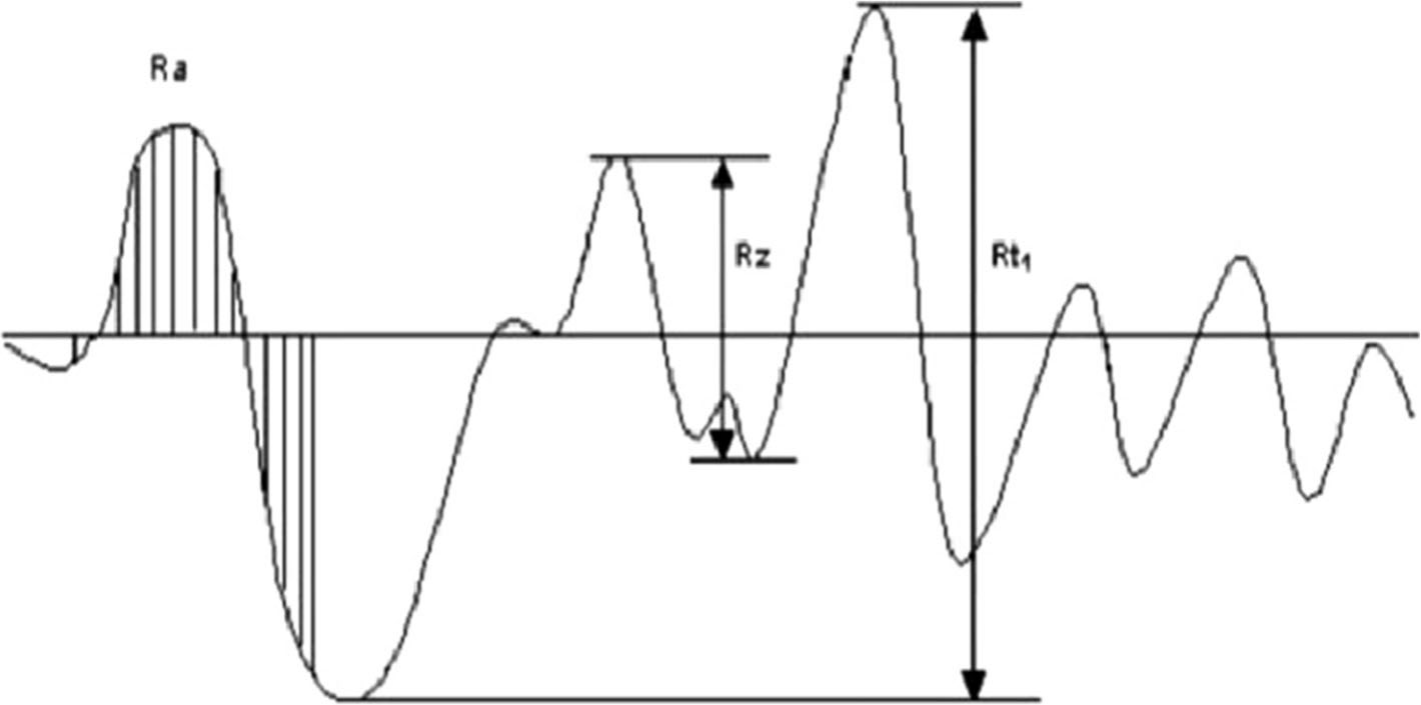
Roughness parameters

The surface roughness is measured using the following three parameters [9]: (Fig. 2)

1. *R*_a_: *R*_a_ is the average roughness. It is the arithmetic mean deviation of the surface valleys and peaks from the center line in the measuring length.
2. *R*_t_: *R*_t_ is the maximum roughness height. *R*_t_ is defined as the maximum peak to valley height over the length of sample.
3. *R*_z_: *R*_z_ is the mean roughness depth. *R*_z_ is the mean vertical space linking the highest peak and the deepest valley of five closest measuring sections.

**Fig. 3:**
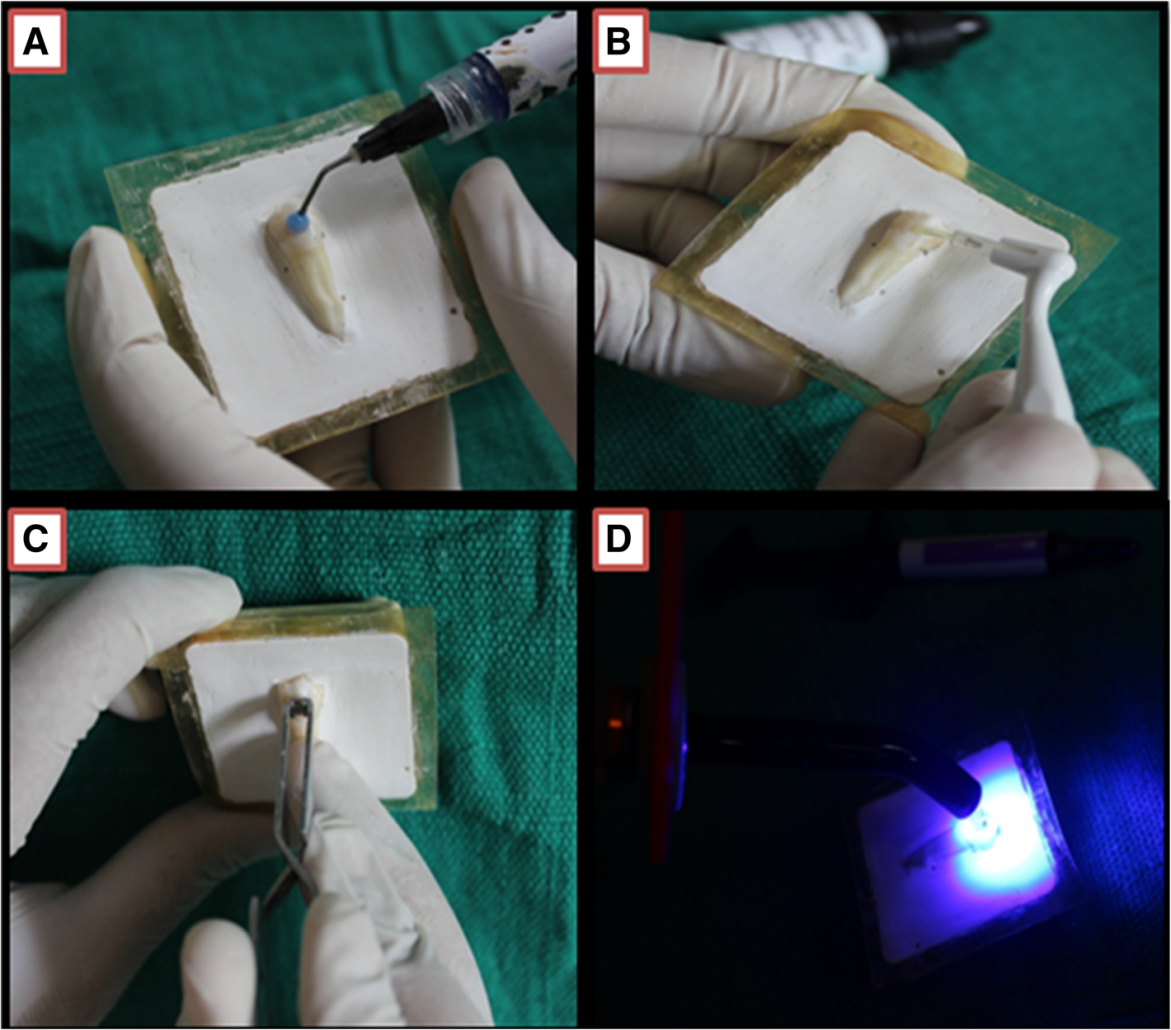
Bonding steps. **a** Etching. **b** Priming. **c** Bonding. **d** Curing

The teeth were polished with pumice slurry and rubber cup. Then they were rinsed with water and dried with compressed air. The buccal surface of the teeth was etched with 37% phosphoric acid for 30 s, rinsed, and air dried (Fig. 3a).

This was followed by primer application on the tooth surface and light cured for 10 s (Fig. 3b). A layer of Vaseline was applied on the mesh of the brackets to avoid composite adhesion to the base of bracket. This will permit easy removal of the bracket with the help of debonding pliers, leaving the entire adhesive on the tooth surface. The adhesive was then applied on the bracket mesh and pushed on the surface of enamel (Fig. 3c).

After removal of excess flash by dental explorer, curing was carried out for total 40 s, from occlusal, gingival, distal, and mesial directions, 10 s each. Wavelength of curing light ranges from 420 to 480 nm (Fig. 3d). The bracket debonding was carried out by gently squeezing the mesial and distal wings with the help of bracket removal pliers.

All burs/wheels/discs were used in low-speed handpiece (10,000–20,000 rpm) with water cooling as per the instructions of the manufacturer. Complete removal of resin was confirmed under dental operating light for visual examination followed by tactile assessment using a dental explorer.

**Fig. 4:**
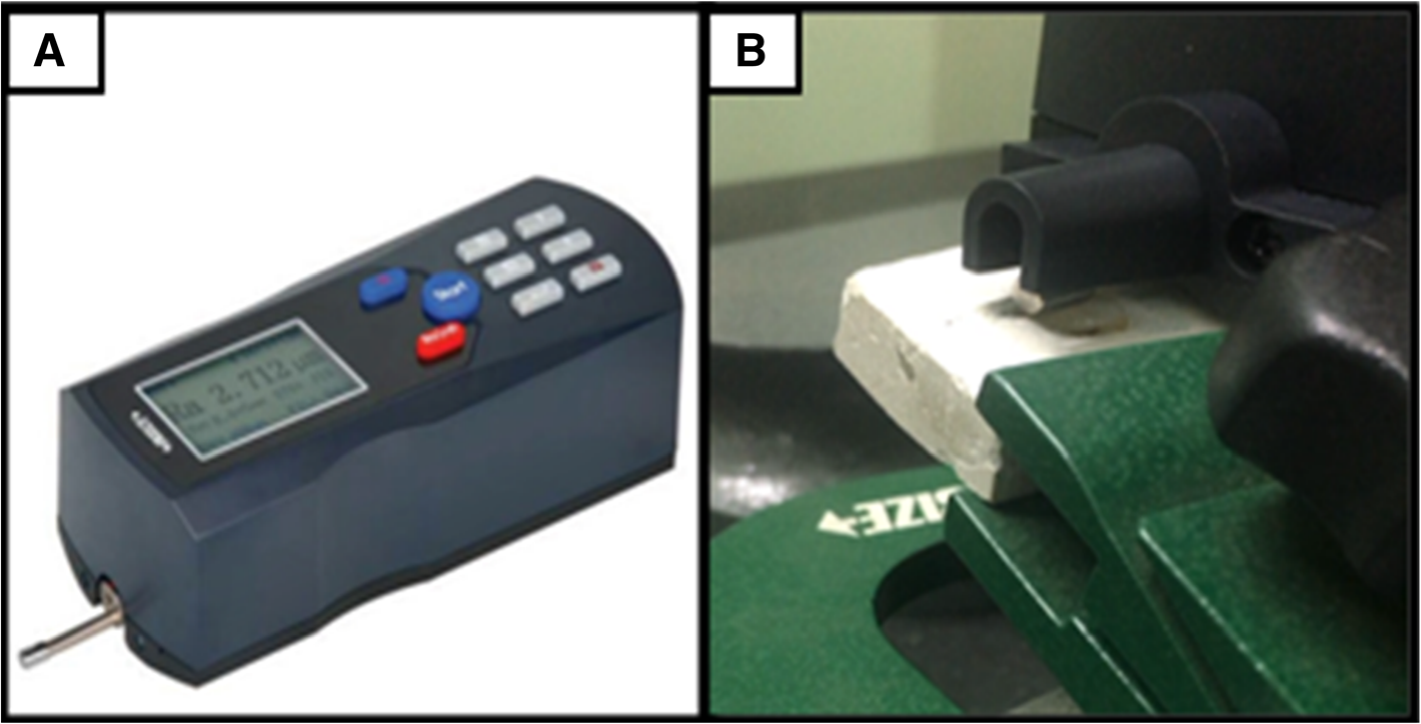
**a** Surface roughness tester. **b** Roughness tester evaluating enamel surface roughness

After residual resin removal with four different methods (Fig. 1), the specimens were subjected to roughness assessment by surface roughness tester (post polishing roughness) (Fig. 4).

Two measurements for each specimen were recorded and mean was calculated.

Out of each group, two specimens were subjected to scanning electron microscopic examination. All bonding, debonding, and adhesive residual removal procedures were carried out by the principal investigator. The data of the above procedures was obtained and subjected to statistical evaluation.

### Scanning electron microscope analysis

The teeth were stored in phosphate-buffered saline (PBS) solution. After drying with air pressure, teeth were mounted on an aluminum stub. Then they were subjected to SEM observation (JEOL, JSM 5610LV, Japan).

Observations were performed at 20 kV and at a working distance of 100 μm with image capturing magnification of × 200. ImageJ Software was used for image analysis. Enamel Damage Index (EDI) was used for assessing enamel surface damage.

ImageJ is an image processing program that can calculate area and pixel value of user defined selections. During the analysis, images were magnified and after 10 mm distance had been defined on the ruler of the photographic setting; the scale was adjusted to pixels/mm for size accordance [11].

EDI [12] includes four scores: score 0 indicates smooth enamel surface without presence of scratches. Perikymata may be seen on enamel surface. Score 1 indicates acceptable enamel surface with fine scattered scratches that involves 1–10% of enamel surface. Score 2 indicates rough enamel surface with several coarse scratches or minor grooves that may involve 11–50% of enamel surface. Score 3 indicates coarse scratches or wide grooves that may involve more than 50% enamel surface. Enamel damage in this score is visible with naked eye.

## Results

### Quantitative observations

No statistically significant difference was found in baseline roughness between four groups with *p* value of 0.666 (*R*_a_), 0.925 (*R*_t_), and 0.702 (*R*_z_).

Comparison of post polishing *R*_a_ using one-way ANOVA test showed that the mean value of group 4 (4.62 μm) was highest, followed by group 1 (3.36 μm), group 2 (3.17 μm), and group 3 (1.99 μm). This difference was statistically significant with *p* value of < 0.001 (Table 2).

**Table 2:** Comparison of difference in *R*_a_ (average roughness) between baseline and post polishing roughness

*R*_a_	Mean baseline roughness	Mean post polishing roughness	Mean difference *R*_a_
Group 1	0.86 μm	3.36 μm	2.50 μm
Group 2	0.95 μm	3.17 μm	2.22 μm
Group 3	0.82 μm	1.99 μm	1.16 μm
Group 4	0.84 μm	4.62 μm	3.78 μm
*p* value	0.666	< 0.001	< 0.001

*R*_a_ average roughness, *μm* micrometer, *p* value probability value

Comparison of post polishing *R*_t_ using one-way ANOVA test showed that the mean value of group 4 (5.36 μm) was highest, followed by group 2 (4.24 μm), group 1 (4.24 μm), and group 3 (3.84 μm). This difference was statistically significant with *p* value of < 0.001 (Table 3).

**Table 3:** Comparison of difference in *R*_t_ (maximum roughness height) between baseline and post polishing roughness

*R*_t_	Mean baseline roughness height	Mean post polishing roughness height	Mean difference *R*_t_
Group 1	1.24 μm	4.24 μm	3.00 μm
Group 2	1.28 μm	4.24 μm	2.96 μm
Group 3	1.26 μm	3.84 μm	2.58 μm
Group 4	1.28 μm	5.36 μm	4.08 μm
*p* value	0.925	< 0.001	< 0.001

*R*t maximum roughness height, *μm* micrometer, *p* value probability value

Comparison of post polishing *R*_z_ using one-way ANOVA test showed that the mean value of group 4 (10.93 μm) was highest followed by group 2 (8.40 μm), group 1 (8.19 μm), and group 3 (7.95 μm). This difference was statistically significant with *p* value of < 0.001 (Table 4).

**Table 4:** Comparison of difference in *R*_z_ (mean roughness depth) between baseline and post polishing roughness

*R*_z_	Mean baseline roughness depth	Mean post polishing roughness depth	Mean difference *R*_t_
Group 1	2.10 μm	8.19 μm	6.09 μm
Group 2	2.02 μm	8.40 μm	6.38 μm
Group 3	2.11 μm	7.95 μm	5.84 μm
Group 4	2.04 μm	10.93 μm	8.89 μm
*p* value	0.702	< 0.001	< 0.001

*Rz* mean roughness depth, *μm* micrometer, *p* value probability value

Post-hoc Tukey test comparing post polishing *R*_a_ showed that the mean value of group 4 (4.62 μm) is highest, followed by group 1 (3.36 μm), group 2 (3.17 μm), and least in group 3 (1.99 μm). This difference is statistically significant with *p* value of < 0.001 (Tables 5, 6, and 7).

**Table 5:** Post-hoc tests for *R*_a_ (average roughness)

Dependent variable	Comparison group	Compared with	Mean difference (unit-μm)	Std. error	*p* value
Baseline *R*_a_	Group 1	Group 2	− 0.09	0.11	0.857
		Group 3	0.04	0.11	0.983
		Group 4	0.02	0.11	0.997
	Group 2	Group 3	0.13	0.11	0.654
		Group 4	0.11	0.11	0.756
	Group 3	Group 4	− 0.02	0.11	0.998
Post polishing *R*_a_	Group 1	Group 2	0.19	0.16	0.627
		Group 3	1.37*	0.16	< 0.001
		Group 4	− 1.25*	0.16	< 0.001
	Group 2	Group 3	1.17*	0.16	< 0.001
		Group 4	− 1.45*	0.16	< 0.001
	Group 3	Group 4	− 2.63*	0.16	< 0.001
Difference *R*_a_	Group 1	Group 2	0.28	0.18	0.417
		Group 3	1.33*	0.18	< 0.001
		Group 4	− 1.28*	0.18	< 0.001
	Group 2	Group 3	1.04*	0.18	< 0.001
		Group 4	− 1.56*	0.18	< 0.001
	Group 3	Group 4	− 2.61*	0.18	< 0.001

*R*_a_ average roughness, *μm* micrometer, *p* value probability value, *statistical significance

**Table 6:** Post-hoc tests for *R*_t_ (maximum roughness height)

Dependent variable	Comparison group	Compared with	Mean difference (unit-μm)	Std. error	*p* value
Baseline *R*_t_	Group 1	Group 2	− 0.04	0.06	0.924
		Group 3	− 0.01	0.06	0.992
		Group 4	− 0.03	0.06	0.951
	Group 2	Group 3	0.02	0.06	0.987
		Group 4	0.005	0.06	1
	Group 3	Group 4	− 0.015	0.06	0.995
Post polishing *R*_t_	Group 1	Group 2	− 0.0004	0.15	1
		Group 3	0.39	0.15	0.062
		Group 4	− 1.12*	0.15	< 0.001
	Group 2	Group 3	0.39	0.15	0.061
		Group 4	− 1.12*	0.15	< 0.001
	Group 3	Group 4	− 1.51*	0.15	< 0.001
Difference in *R*_t_	Group 1	Group 2	0.04	0.17	0.996
		Group 3	0.41	0.17	0.085
		Group 4	− 1.08*	0.17	< 0.001
	Group 2	Group 3	0.37	0.17	0.141
		Group 4	− 1.12*	0.17	< 0.001
	Group 3	Group 4	− 1.50*	0.17	< 0.001

*R*_t_ maximum roughness height, *μm* micrometer, *p* value probability value, *statistical significance

**Table 7:** Post-hoc tests for *R*_z_ (mean roughness depth)

Dependent variable	Comparison group	Compared with	Mean difference (unit-μm)	Std. error	*p* value
Baseline *R*_z_	Group 1	Group 2	0.07	0.09	0.824
		Group 3	− 0.01	0.09	1
		Group 4	0.06	0.09	0.903
	Group 2	Group 3	− 0.08	0.09	0.765
		Group 4	− 0.01	0.09	0.998
	Group 3	Group 4	0.07	0.09	0.857
Post polishing *R*_z_	Group 1	Group 2	− 0.20	0.17	0.633
		Group 3	0.23	0.17	0.527
		Group 4	− 2.73*	0.17	< 0.001
	Group 2	Group 3	0.44	0.17	0.059
		Group 4	− 2.53*	0.17	< 0.001
	Group 3	Group 4	− 2.97*	0.17	< 0.001
Difference in *R*_z_	Group 1	Group 2	− 0.28	0.17	0.384
		Group 3	0.24	0.17	0.515
		Group 4	− 2.80*	0.17	< 0.001
	Group 2	Group 3	.53*	0.17	0.019
		Group 4	− 2.51*	0.17	< 0.001
	Group 3	Group 4	− 3.04*	0.17	< 0.001

*R*_z_, mean roughness depth, *μm* micrometer, *p* value probability value, *statistical significance

### Qualitative observations

Scanning electron microscopic (SEM) observations allowed identifying both the adhesive residuals and the enamel damage.

**Fig. 5:**
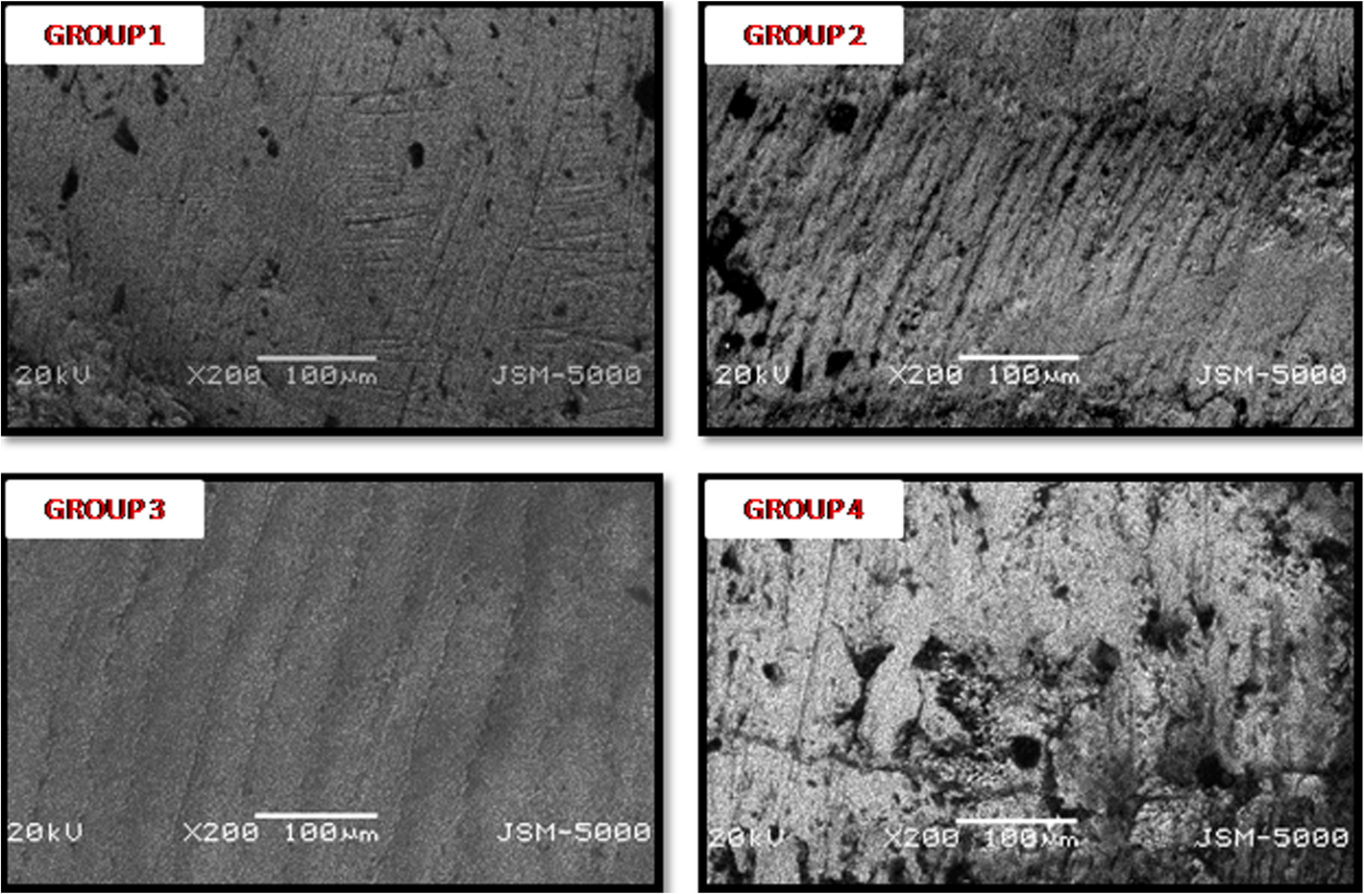
Representative SEM micrographs of enamel surfaces at 200× magnification after clean-up with: **1**-One Gloss system; **2**-Enhance and Pogo system; **3**-Stainbuster; **4**-Soflex disc and wheels

EDI score 0 was observed in group 3 (Stainbuster group) which showed smooth surface without presence of scratches. Score 1 was noted in group 2 (Enhance and Pogo system) which showed acceptable surface with fine scratches. Score 3 was noted in group 1 and group 4 with coarse scratches and wide grooves (Fig. 5) (Tables 8 and 9).

**Table 8:** Enamel damage scores of four groups

Groups	Enamel damage index score
Group 1:	
Sample 1	3
Sample 2	3
Group 2:	
Sample 1	0
Sample 2	0
Group 3:	
Sample 1	1
Sample 2	1
Group 4:	
Sample 1	3
Sample 2	3

**Table 9:** Chi-square test for enamel roughness evaluation (SEM)

Group * score cross tabulation						
			Score			Total
			.00	1.00	3.00	
Group	Group 1	Count	0	0	2	2
		% within score	0.0%	0.0%	50.0%	25.0%
	Group 2	Count	2	0	0	2
		% within score	100.0%	0.0%	0.0%	25.0%
	Group 3	Count	0	2	0	2
		% within score	0.0%	100.0%	0.0%	25.0%
	Group 4	Count	0	0	2	2
		% within score	0.0%	0.0%	50.0%	25.0%
Total		Count	2	2	4	8
		% within score	100.0%	100.0%	100.0%	100.0%

Fishers exact value of 10.064 and *p* value of 0.029

## Discussion

Correct bonding and debonding techniques play an important role in orthodontics. Many factors affect these procedures; the most important among them are the type of adhesive used for bonding, the instruments used for bracket debonding, and the finishing and polishing methods of adhesive resin removal [13].

With evolution of composite resin and adhesive systems, more effective bonding between enamel and resin can be achieved resulting in fewer brackets debonding rate. But, due to this increased adhesion of resin to enamel surface, removal of resin after debonding becomes more troublesome. So, the technique used for residual resin removal plays important role to avoid enamel surface damages, such as enamel cracks, rougher enamel surface, wear of enamel, overheating of the teeth, and pulpal damage [14].

In our study, no statistically significant difference in baseline surface roughness was noted between One gloss group, Enhance system group, Stainbuster group, and Soflex disc and wheels group. On the other hand, finishing instruments affected the surface roughness parameters. Higher roughness values were obtained with use of the Soflex group than other three groups (*p* < 001).

Our results showed that Soflex disc group created roughest enamel surface in comparison to other three groups. Results were in accordance with the results of Challa et al. [15], who assessed the effectiveness of five resin removal methods including tungsten Carbide burs (TCB), Discs (Sof-Lex), One step system (PoGo), and combination of carbide bur with multistep and one step polishing systems (TCB + Sof-Lex + pogo). Scanning electron microscopic results showed that enamel surface was near to original in samples finished with One step system (PoGo) followed by Sof-Lex dics.

Howell [16] in his study observed that Sof-Lex discs followed by slurry of pumice led to roughest enamel surface. Michele vidor [17] recommended using Enhance finishing tip followed by aluminum oxide polishing rather than Soflex discs as more enamel damage was observed with Soflex system.

Didem Atabek [7] evaluated enamel surface roughness after debonding using Enhance and pogo system, Stainbuster bur, and Soflex discs. Profilometric analysis suggested that smoothest enamel surface was obtained with Enhance system and roughest enamel surface was achieved using Soflex discs.

Brijesh in their in vitro study compared Enhance and Pogo system, One gloss system, and Soflex spiral wheels. Their results suggested that Enhance and Pogo micro polisher were better in creating smoother enamel surface than One gloss system and Soflex discs [18].

In the present study, SEM was used to assess enamel surface configurations. This method cannot provide a quantitative assessment. It is only used as a supportive tool with quantitative assessment methods [19].

SEM micrographs showed that Stainbuster bur seemed to be very efficient way to clean the surface. This qualitative result of smoothest surface achieved with Stainbuster bur agreed with our quantitative result. One gloss system and Soflex discs were the most hazardous techniques to the enamel surface. Enhance and pogo system was less destructive to enamel surface than One Gloss system and Soflex disc and wheels.

In the current study, surface roughness tester was used for assessing enamel surface roughness quantitatively. The surface roughness tester (Insize, ISR-C100) can measure up to 16 different parameters. The tester has a probe position indicator that helps in accurate identification of location. Display window shows roughness values, profile, and curves. The instrument can get connected with printer via Bluetooth [20].

All measured roughness parameters (*R*_a_, *R*_t_, *R*_z_) for Sof-Lex disc and wheels were statistically higher than other finishing and polishing systems employed in this study. While all the roughness values were lowest for stainbuster group indicates smoothest enamel surface among all four groups.

The differences in roughness after finishing and polishing among the techniques also depend on patterns of particle size and their organization within the resin matrix of respective bur/wheel/disc. Aluminum oxide disks have limitations because of their shape, which make them difficult to use efficiently in posterior teeth [21].

Bicakci [22] used high speed burs without water cooling. They noticed heating in the pulp chamber resulting in vascular hyperemia and occasional breakage of odontoblasts was seen. This is a transient reversible condition. Damage of pulp gets repaired within about 20 days. It was recommended to remove most of the residual resin under water cooling and turning the water cooling off during removal of last resin layer, so that it helps in distinguishing between enamel surface and resin remnants, thereby preventing further enamel damage and loss. Therefore, in our study, water cooling was used initially to remove bulk of composite and last layer of resin was removed without water coolant.

Speed of hand piece is one of the important issues while removing adhesive resin with hand piece. A low-speed rotary instrument creates additional vibrations and uncomfortable for patients [23]. Risk of pulpal damage increases with low-speed instrument [24]. It is noted that low-speed instrument created irregular enamel surface but the natural enamel itself also showed slightly repetitive and spiky enamel [23]. It was found that effective adhesive removal was achieved with low-speed burs than high-speed burs due to the fact that both the depth and the area of the residual resin layer were significantly lower after using low-speed burs (*p* value < 0.05). Bishara et al. [24] observed that enamel loss was less with low-speed burs than high-speed burs. For such reasons, slow speed hand piece was used in our study.

Generation of aerosols is another demerit of residual resin removal with rotary instrument. Jonke E et al. [25] in their study observed that after ceramic bracket debonding and various cleanup methods, aerosols produced during composite grinding can act as endocrinological disruptors.

Debonding and adhesive resin removal techniques are operator-dependent procedures. Thus, the results may probably differ among operators. So as to lessen this inaccuracy, just one operator carried out all the clinical procedures in our study. Results of the current study showed that post clean-up roughness (post polishing roughness) was more than the prebonding enamel roughness (baseline roughness) in all the four groups. This implies that no resin removal method was able to completely restore the enamel surface roughness to its original form [26].

Our study has some limitations and warrant future studies to combat the same. Latest methods such as confocal laser microscopy and atomic force microscopy (AFM) are being used to obtain 3D data of enamel roughness that will help in gaining more clear information regarding the amount of enamel loss caused due to various resin removal methods.

Second limitation of our study is in vitro study. Our study being in vitro, the result of this study cannot be directly applied in clinical situations. Factors such as saliva, oral hygiene, temperature, and pH can also affect our results. Future in vivo studies are required to confirm our results and clinical implementation [27].

## Conclusions

1. Stainbuster bur created the smoothest enamel surface that was close to the natural enamel followed by Enhance system, One gloss system, and Soflex disc and wheels in terms of post polishing average roughness (*R*_a_).
2. Post polishing maximum roughness height (*R*_t_) and mean roughness depth (*R*_z_) were least in Stainbuster group and highest in Soflex disc and wheels group.
3. Scanning electron microscopic (SEM) examination showed that Stainbuster bur (group 3) was least damaging to enamel surface among all four methods.

## Abbreviations

EDI: Enamel Damage Index
SEM: Scanning electron microscopy
μm: Micrometer
